# Informatics-Based Design of Virtual Libraries of Polymer Nano-Composites

**DOI:** 10.3390/ijms26157344

**Published:** 2025-07-30

**Authors:** Qinrui Liu, Scott R. Broderick

**Affiliations:** Department of Materials Design and Innovation, University at Buffalo, Buffalo, NY 14260, USA

**Keywords:** polymer matrix nano-composites, quantitative structure–activity relationship (QSAR), materials informatics, electrical conductivity

## Abstract

The purpose of this paper is to use an informatics-based analysis to develop a rational design approach to the accelerated screening of nano-composite materials. Using existing nano-composite data, we develop a quantitative structure–activity relationship (QSAR) as a function of polymer matrix chemistry and nano-additive volume, with the property predicted being electrical conductivity. The development of a QSAR for the electrical conductivity of nano-composites presents challenges in representing the polymer matrix chemistry and backbone structure, the additive content, and the interactions between the components while capturing the non-linearity of electrical conductivity with changing nano-additive volume. An important aspect of this work is designing chemistries with small training data sizes, as the uncertainty in modeling is high, and potentially the representated physics may be minimal. In this work, we explore two important components of this aspect. First, an assessment via Uniform Manifold Approximation and Projection (UMAP) is used to assess the variability provided by new data points and how much information is contributed by data, which is significantly more important than the actual data size (i.e., how much new information is provided by each data point?). The second component involves assessing multiple training/testing splits to ensure that any results are not due to a specific case but rather that the results are statistically meaningful. This work will accelerate the rational design of polymer nano-composites by fully considering the large array of possible variables while providing a high-speed screening of polymer chemistries.

## 1. Introduction

The design of polymer nano-composites represents a highly multivariate design problem because of the large array of possible descriptors for the polymer matrix, the nano-additive, and the interactions between the components [1,2,3]. Because of this multi-dimensionality, an informatics-aided approach to the design of polymer nano-composites will accelerate the design of new nano-composites [4,5]. We previously identified structure–property relationships in polymer systems [6,7,8,9]. Here we use informatics to identify structure–property relationships via the establishment of a QSAR, which can provide a high-speed screening of new chemistries. The purpose of this paper is to develop a template of the approach to very rapidly computationally generating hundreds of combinations of chemistries and microstructures, after which electronic structure calculations and experiments can be used to refine issues with the prediction.

Polymer matrix composites (PMCs) are of particular interest because of their beneficial mechanical and electrical properties [10,11,12]. The properties of PMCs are altered by changing polymer chemistry, additive type, size or geometry, and processing [13,14]. However, an improvement in the property of interest such as electrical conductivity is often met with the worsening of other properties of interest. For example, in aerospace applications, large ductility is desired in order to form the body of an airplane while also accounting for electrical conductivity, as occurs in lightning strikes. The challenge is to find some combination of matrix and additive which can improve a property without resulting in a negative impact on other properties [15,16,17]. One approach to addressing this challenge is to create a significant amount of data via high-throughput experimentation and to then search through the results for conditions meeting the design criteria. We instead use an informatics-based approach, which can then be extended to the development of a series of QSAR models for identifying crucial molecular structure descriptors and providing a guide to nano-composite design.

The concept of QSARs is taken from biology [18,19], and for the study of polymers, we define the activity instead as a property. A QSAR defines the relationship between molecular structures, the nano-additive, and properties [20]. Beyond providing a high-speed modeling approach, the QSAR also provides chemically meaningful relationships by defining how the individual molecular units impact properties [21,22]. Therefore, the QSAR helps in understanding the reason why the composite has its property in terms of chemistry, leading to a rational design strategy as opposed to an Edisonian design strategy [23,24]. QSARs can be developed for multiple properties so that the molecular units leading to improvement in numerous and possibly inversely related properties can be identified, and new chemistries can be suggested for experimental validation [25,26,27,28].

The objective of this work is to build a model connecting chemical descriptors and additive volume with electrical conductivity. Such a model then allows for the calculation of the electrical conductivity of any polymer chemistry or any PMC with carbon nanofiber (CNF) as the additive. The larger purpose of this work is to build a framework for developing QSARs for multiple properties as a function of molecular structure and composition and additive volume. Finally, the design of materials given a small dataset is considered. This aspect is especially important, as we often deal with small data sizes in materials science. However, this paper serves to argue that the role of data-driven analyses is still useful in such cases, as long as the increased uncertainty and added assessment of the models and data are addressed. In this paper, these important considerations are discussed.

## 2. Results

### 2.1. Attribute Testing

An important consideration in PMC design is the inter-relationships between properties. Of specific interest for developing a QSAR for electrical conductivity is investigating the change in impact for the molecular descriptors of a neat polymer resin versus a PMC. The reasons behind the interest in comparing the impact of descriptors on electrical conductivity are two-fold. First, if the relative impact of the descriptors on electrical conductivity remains consistent, then the model would be considered significantly robust, and this would suggest that the model does not only capture the effect of the additive while using the molecular descriptors to over-fit the model. Secondly, the objective in developing a QSAR is to screen for not only additive content but also new polymer chemistries. Therefore, if the relationships between property and molecular descriptors remain consistent, we conclude that the interactions due to additives do not cause changes in the chemistry–property relationships, and we can indeed use the QSAR to screen any applicable polymer chemistry regardless of additive content.

**Figure 1:**
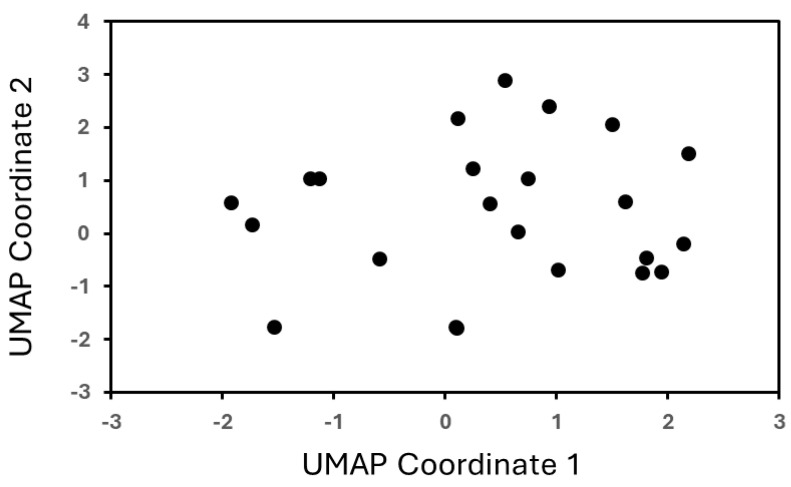
A representation of the data. This analysis serves to explore the variety of data and information contributed by each descriptor. This type of analysis is critical while exploring small data, as the variability contributed by the data is much more important than the size of the data. From this analysis, we see that the data is fairly evenly distributed, and therefore even though we have small data, the data included largely contributes unique information.

An obvious challenge in the data-driven design of materials is the small data size. An important consideration in design with small data, however, is not the size of the data but rather the diversity of information captured by the data. For this purpose, we employed Uniform Manifold Approximation and Projection (UMAP) to visualize the feature space in a two-dimensional space. UMAP projects the high-dimensional non-linear complexity of the polymer chemistries onto a plane, facilitating an intuitive understanding of the material space coverage. The objective of this analysis is to explore the distribution of the data. In the case of insufficient representation, the data would largely be clustered together. However, as seen in Figure 1, the data is well distributed across the UMAP space.

**Figure 2:**
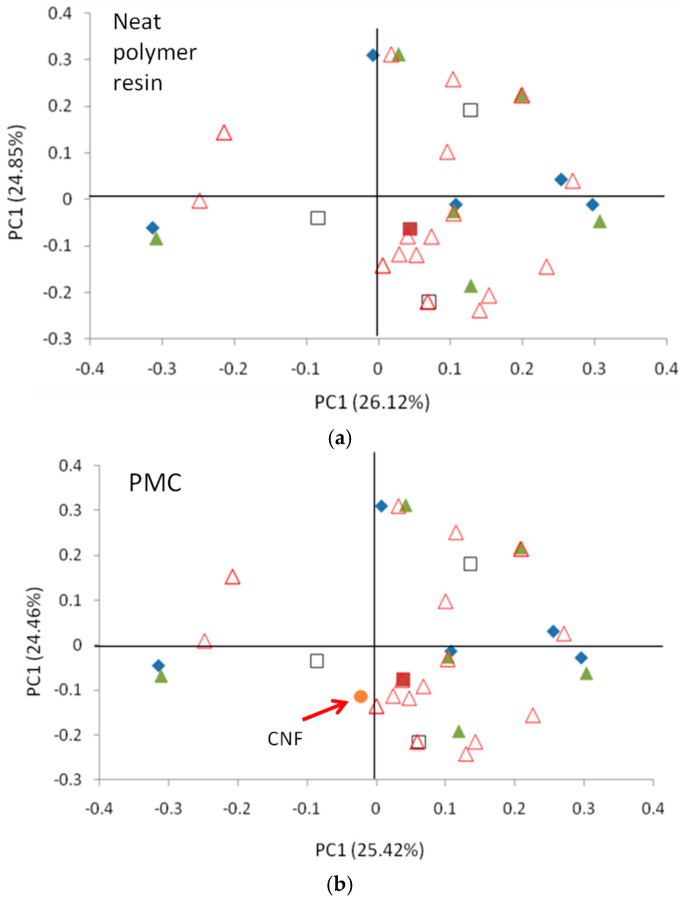
A loading plot comparing the relationships between descriptors on electrical conductivity for polymers (**a**) versus PMCs (**b**). The relationships are similar, which demonstrates the ability of informatics to capture high-dimensional relationships between the polymer, the additive, and the property. To aid in comparison, different symbols are used for different groups of molecular descriptors, in addition to the filled red square representing electrical conductivity and the filled orange circle representing carbon nanofiber volume. The symbols are as follows: a filled blue diamond for composition, a filled green triangle for backbone atoms, an open black square for side groups, and open red triangles for specific bonding descriptions. The arrow is pointing to the carbon nanofiber volume descriptor, which is the added descriptor in the PMC analysis.

In Figure 2, we compare the loading plot with electrical conductivity for (a) neat polymer resins and (b) PMCs with various amounts of carbon nanofiber additive. In this figure, we find that there is relatively little change in the qualitative relationships between the molecular structure descriptors and electrical conductivity. Based on the logic discussed, we identify our QSAR as being sufficiently robust and capturing the effect of the polymer and the nano-additive independently.

To further describe the relationships between the polymer matrices and the properties, the specific PC values of the molecular descriptors in Figure 2 are listed in Table 1. From this table, the relationship between composition and molecular structure with the specific properties can be identified.

### 2.2. QSAR Development and Application

**Figure 3:**
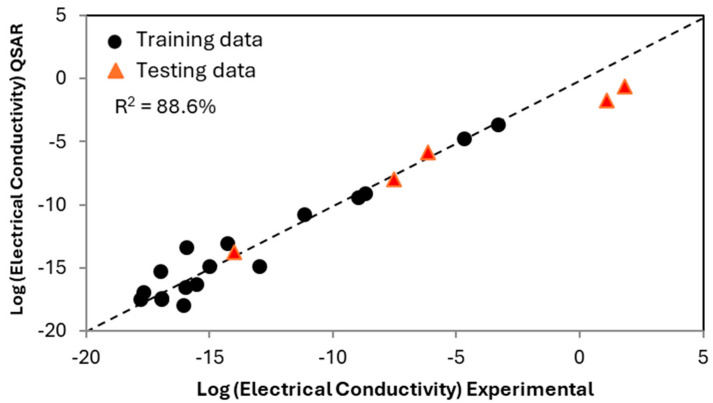
The predicted electrical conductivity of neat polymer resins and PMCs containing a thermoplastic matrix with a carbon nanofiber additive. The equation is for electronic conductivity as a function of polymer chemistry, backbone structure, and volume additive. Based on this QSAR, the electrical conductivity of any volume of carbon nanofiber in any polymer matrix comprising the modeled molecular units can theoretically be predicted. The triangles display predicted PMCs not used in developing the QSAR.

Having tested the robustness of the appropriate descriptors and verifying that the model will not be overly dominated by the nano-additive, a QSAR was developed as a function of polymer matrix composition and structure and nano-additive volume, with the nano-additive limited to carbon nanofibers. The result of the QSAR as compared to experimental data is shown in Figure 3. Seventeen PMCs and neat polymer resins were used to develop the QSAR (displayed as circles), while five additional PMCs were not included in the training dataset but were used instead for validation (displayed as triangles). The relative agreement between the predicted and actual values for the PMCs suggests that the QSAR can be used to predict new PMCs and provides a high-speed screening of polymer chemistries.

In Figure 3, each point represents either a PMC or a neat polymer resin, with the majority of points in the low-electrical-conductivity cluster being neat polymer resins. The horizontal axis represents the experimentally measured values of electrical conductivity, while the vertical axis represents the electrical conductivity predicted with informatics. As the predictions of the PMCs capture the trend well, the interactions between the polymer matrix and additive are described within the existing knowledge base, and the QSAR captures these interactions without explicitly defining them. The accuracy of the prediction of PMC properties demonstrates that electrical conductivity is directly impacted by individual units within the polymer matrix and the interaction between the nano-additive and individual chemical units. Also of note, the materials with the highest electrical conductivity were kept as training data. The lower accuracy for these is due to the challenges in model extrapolation; however, qualitatively, the predictions are still reasonable, showing the robustness of the model.

To test the dependency on the selection of training versus testing data, we ran computations with multiple random cuts of the data. This step is particularly important given the small dataset. The results of the different cuts are provided in Table 2. Each row corresponds to a different 80/20 split in training versus testing data. The R^2^ value is for the overall data, while the RMSE values provide a more relevant metric given the small dataset. From the different cuts, we see that the data, in general, is over-fit but not to an unusable level. Further, the results are not sensitive to the training/testing split. One result (split 5) has much lower accuracy; however, this result is due to the split including points in testing exclusively at the edges of the data and therefore reflects the challenges in model extrapolation.

**Figure 4:**
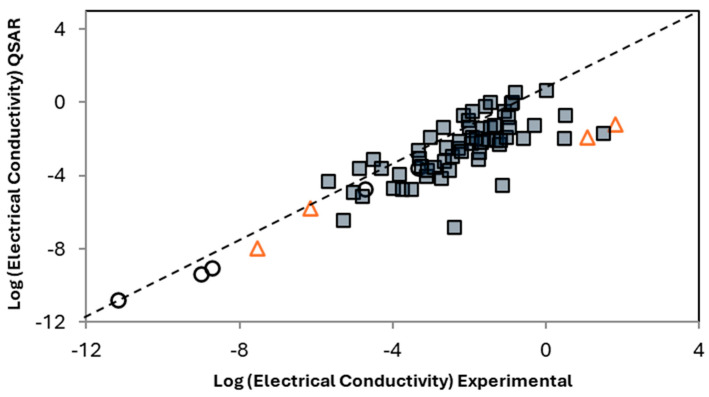
The application of the developed model to additional previously unseen data for PMCs containing CNF. The gray squares are new points added after the model was built for further testing, while the black circles and red triangles correspond with the training and testing points, respectively, in Figure 3. For ease of visualization, the region shown focuses on CNFs and does not include the neat polymer resins. The model captures the trend with electrical conductivity relatively well, providing additional confidence in the robustness of the model, even with the small initial training data.

As stated in the Introduction, a focus of this work was the data-driven design and improved understanding of materials when dealing with small data sizes. However, there are still significant challenges with uncertainty and assessing any level of future confidence in the results. For this purpose, the model developed and applied was applied to additional data [29,30,31,32,33,34] found after the model was built and was therefore entirely unseen and serves fully as a test. The results with this added data are shown in Figure 4. These results demonstrate that the model is not only applicable for the small dataset but can indeed be used to at least screen potential systems of interest. The testing of significantly more points comes with increased uncertainty in the results, but it also clarifies the utility of the model as a screening tool. That is, prior to experimental testing, this model (specifically in the case of electrical conductivity shown here) can be used to screen potential candidates and identify the most promising candidate, thereby reducing the number of unnecessary experiments and increasing the probability of success. Given the limited dataset size and scope of included materials, this study serves as a preliminary demonstration of the feasibility of informatics-based modeling approaches in the design of polymer nano-composites.

## 3. Discussion

**Figure 5:**
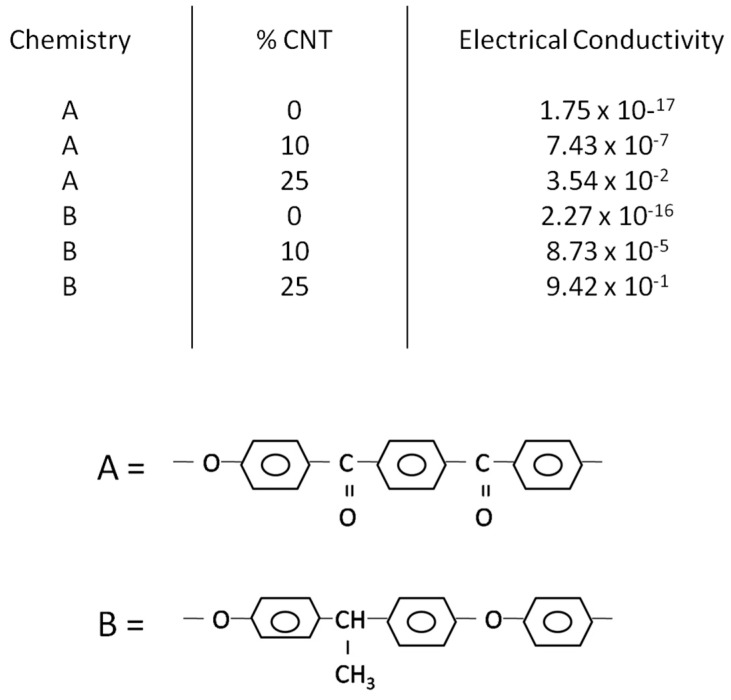
A demonstration of using a QSAR to predict electrical conductivity as a function of molecular chemistry and microstructure for both a known polymer (PEKK) and a virtual polymer. This development of a virtual nano-composite library has implications for identifying both new promising polymers and the underlying driving physics.

Having developed the QSAR model, we demonstrate how it may be utilized to develop a virtual library of polymer chemistries. In Figure 5, the rapid prediction of electrical conductivity for polyether ketone ketone (PEKK) and a virtual polymer is shown. The ability to process any virtual polymers is not considered at this point, but rather the focus is on how a QSAR is able to create a large virtual library of nano-composite data rapidly. Using a QSAR, nano-composites showing significant promise can then be further explored with experimentation and electronic structure calculations to refine issues and further describe the underlying physics.

This model for linking matrix chemistry and additive with a property does not only allow for the prediction of the property for a large number of systems but also provides the chemistry–additive relationships. Based on the calculation, the descriptors which impact the property the most can be identified. For the descriptors included, a nitrogen atom bonded to three carbon atoms is the most inversely correlated with electrical conductivity, with a carbon atom in a benzene ring and also being bonded to a nitrogen atom being the second most inversely correlated. A benzene ring as a side group and a backbone CH_2_ group are the most correlated molecular descriptors with electrical conductivity. The volume percent of carbon nanofibers is, in general, approximately four times more important in impacting electrical conductivity than any polymer chemistry.

Additionally, a comparison of similar groups can provide interesting new physical findings linking polymer chemistry with electronic conductivity. For example, a backbone N atom bonded to a H atom, in general, significantly improves electrical conductivity, while a backbone N atom bonded instead to a non-backbone C atom significantly reduces electrical conductivity. Similarly, a backbone C atom bonded to two H atoms increases electrical conductivity generally, while a backbone C atom double bonded to an O atom decreases electrical conductivity. Also, a backbone benzene ring bonded to a backbone O or N atom reduces electrical conductivity, while a backbone benzene ring bonded instead to a C atom increases electrical conductivity. By identifying such relationships, the developed QSAR can be used to identify interesting relationships between changing chemistry and property, beyond just providing a tool for modeling and screening new nano-composites.

This work intentionally constrained the analysis to a consistent processing context to isolate the effects of polymer composition and filler content. However, we recognize that critical factors such as filler dispersion quality, agglomeration, polymer chain alignment, and void fraction can substantially impact electrical conductivity. The absence of explicit descriptors for these processing-related variables represents an important limitation that should be addressed in future studies. Additionally, the model does not explicitly incorporate descriptors for filler network formation and percolation dynamics, which are known to play an important role in forming conductive pathways in nano-composites.

Accordingly, our future work will focus on expanding the dataset to cover a broader range of polymer–nanofiller systems, incorporating processing-related descriptors, and validating models by using larger, more diverse experimental datasets.

## 4. Materials and Methods

The difficulty of mathematically linking polymer chemistry, nano-additive content, and properties involves three challenges that we discuss here: identifying the aspects of polymer chemistry leading to improved properties, incorporating changes in properties and relationships due to the addition of nano-additives, and considering the changes in correlated properties when designing for a single property. Actual models may incorporate alternative property metrics depending on specific application requirements. In this paper, we mathematically link molecular structure descriptors with electrical conductivity, identify how the addition of nano-additives changes these relationships, model the electrical conductivity for a “virtual” nano-composite, and discuss how this work allows for an improvement in nano-composite properties without the expense of worsening other property values.

The informatics approach employed in this work is partial least squares (PLS) [35,36,37,38,39,40]. In PLS, training data is converted to a data matrix with orthogonalized axes, which are based on capturing the maximum amount of information in fewer dimensions. The relationships discovered in the training data can be applied to a test dataset based on the projection of the data onto a high-dimensional hyperplane within the orthogonalized axis system. With PLS, the properties of the composites can be modeled as a function of the chemical and additive descriptors independent of each other. Typical linear regression models do not properly account for the co-linearity between the descriptors, and as a result, the isolated impact of each descriptor on the property cannot be accurately identified [41,42,43,44]. However, by projecting the data onto a high-dimensional space defined by axes which comprise a linear combination of the composite descriptors and are also orthogonalized, the impact of the descriptor on the property can be identified independent of all other descriptors. Therefore, PLS is used here to identify chemistry–additive–property relationships, which can then be used to develop QSARs to model new PMC systems accurately and robustly.

The dataset used in this study was collected from published literature sources focused on thermoplastic polymer matrices with carbon nanofiber additives. To ensure consistency and minimize variability from processing conditions, only studies employing comparable dispersion techniques and curing procedures were included.

**Figure 6:**
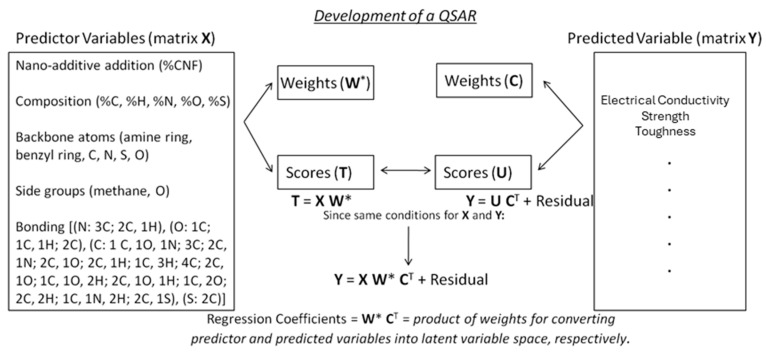
A description of the data used in this analysis and the logic for developing a QSAR. The polymer matrix is described by a variety of descriptors consisting of composition, molecular units and structure, and bonding. PLS operates by performing separate singular value decomposition-like analyses on the predictor matrix and the predicted matrix, outputting the weights needed to convert the matrix to latent variable space and the values of the nano-composites in latent variable space (scores). For example, T = X W* indicates that the scores equals the predictor variables times the weights (W*). The notation of * indicates the collective influence across all of the variables. Since the nano-composite chemistries are the same in both matrices, we substitute the score matrix of the predictor variables for the score matrix of the predicted variables, thus allowing us to connect the predicted variables with the predicted variables based on the regression coefficients defined by a combination of the two weight matrices.

The dataset representing a portion of the existing knowledge base represented in the literature contains numerous properties, but here we limit the property prediction to electrical conductivity. Electrical conductivity is the predicted variable, while the chemical descriptors and volume of additive are the predictor variables. We focus only on thermoplastic polymer resins containing carbon nanofibers under similar processing conditions. The number of descriptors used here to describe electrical conductivity is 22, with the descriptors being multiple chemical descriptors and additive volume, with the chemistries selected to represent chemical diversity. These descriptors were selected to capture relevant variations in polymer elemental composition, backbone and side-chain structures, and bonding mechanisms, as these features are hypothesized to strongly influence conductive pathways. The selection aimed to provide a comprehensive representation of chemical diversity for the polymer matrices studied. Figure 6 illustrates the data considered in this work, as well as defining the matrices used in PLS mathematics. While the term QSAR suggests relationships between structure and activity, the specific relationship in this case is that between molecular chemistry and microstructure with properties.

The following figure also serves to clearly differentiate PLS from related algorithms such as principal component regression (PCR). PCR performs a principal component-based dimensionality reduction on the entire dataset (predictor and predicted variables), and linear regression is then performed on the data in this principal component space. PLS instead performs two principal component-like dimensionality reductions: one on the predictor variables and a separate one on the predicted variables. The various calculated matrices are then combined to form a regression model.

The microstructure is represented by nano-additive volume, with the nano-additive being carbon nanofibers. QSARs can be developed by linking the polymer chemistry and microstructure to numerous properties. In this paper, we develop a template for developing these QSARs by modeling electrical conductivity. The values of these descriptors for a few chemistries are shown in the Supplementary Materials, demonstrating the complexity of the problem. The chemical descriptors were selected to provide a full description of the polymer chemistries. Certain properties, such as T_g_ and density, have been demonstrated as being predicted based only on the chemical descriptors [45,46,47,48]. However, the problem of predicting electrical conductivity for composites is more challenging for several reasons. First, the prediction based only on chemical descriptors is not sufficient because we need to account for additives also. Additionally, no clear relationship between chemical components and electrical conductivity has ever been demonstrated. Finally, and most significantly, the relationship between electrical conductivity and additive content is non-linear [49,50].

## 5. Conclusions

This work developed a QSAR for nano-composites and demonstrated how a QSAR can be used to create a virtual library of polymer nano-composites. In this work, we were able to identify subtle molecular differences that significantly impact electrical conductivity. Additionally, we showed that the relationships between electrical conductivity and the molecular units are consistent for either a neat polymer resin or a PMC, indicating that our model is sufficiently robust in capturing the interaction between units. Having identified the relationship between the possible molecular descriptors and electrical conductivity, additional QSARs can be developed for properties which were shown to be inversely correlated or uncorrelated to electrical conductivity. With these additional QSARs, common molecular units and new chemistries with improved properties can be identified.

## Figures and Tables

**Table 1:** The values of the descriptors in the loading plots in Figure 2. In this table, BB indicates a backbone atom, NBB indicates a non-backbone atom, and the atoms in parentheses indicate the bonding atoms.

	PC1 (Figure 2a)	PC2 (Figure 2a)	PC1 (Figure 2b)	PC2 (Figure 2b)
%C	−0.109	0.282	0.297	−0.012
%H	0.165	−0.252	−0.313	−0.062
%N	−0.248	−0.110	−0.007	0.310
%O	−0.067	0.125	0.254	0.042
%S	−0.019	0.099	0.108	−0.012
BB Amine	−0.260	−0.068	0.197	0.225
BB Benzyl	−0.006	0.101	0.307	−0.047
BB C	−0.246	0.134	−0.308	−0.084
BB N	0.218	0.095	0.028	0.312
BB S	−0.053	0.296	0.105	−0.025
BB O	0.182	−0.240	0.128	−0.186
NBB Methane	0.163	0.070	0.070	−0.220
NBB O	−0.218	−0.095	0.128	0.192
N (C,C,C)	−0.245	0.135	0.198	0.224
N (C,C,H)	−0.005	−0.308	−0.215	0.144
O (C)	−0.250	−0.079	0.103	0.258
O (C,H)	0.180	0.037	0.005	−0.141
O (C,C)	0.211	0.083	0.140	−0.238
C (N,O,C)	−0.256	−0.081	0.017	0.311
C (1.5C,1.5C,C)	−0.136	0.228	0.269	0.040
C (1.5C,1.5C,N)	−0.245	0.135	0.198	0.224
C (1.5C,1.5C,O)	0.174	0.139	0.153	−0.205
C (1.5C,1.5C,H)	0.060	0.289	0.233	−0.144
C (C,H,H,H)	0.219	0.092	0.068	−0.219
C (C,C,C,C)	0.219	0.092	0.068	−0.219
C (C,C,O)	0.061	0.050	0.073	−0.079
C (C,O,H,H)	0.148	0.002	0.028	−0.117
C (C,C,O,H)	0.180	0.037	0.005	−0.141
C (C,O,O)	0.051	−0.022	0.040	−0.079
C (C,C,H,H)	−0.005	−0.308	−0.248	−0.002
C (C,N,H,H)	−0.005	−0.308	−0.215	0.144
C (1.5C,1.5C,S)	−0.001	0.102	0.103	−0.030
S (C,C)	−0.104	0.062	0.095	0.102

**Table 2:** R^2^ values for the total data resulting from the model and training and testing RMSE. The results of this analysis show that the model’s accuracy is not overly dependent on the selection of the 80/20 split in chemistries for training/testing. This step is important given the small data size, but it also demonstrates the robustness of the model, particularly given the relatively large descriptor size.

Split	Overall R^2^	Training RMSE	Testing RMSE
1	88.6%	1.05	5.26
2	84.9%	3.13	5.67
3	89.1%	4.25	2.24
4	87.4%	3.07	5.37
5	56.0%	7.05	10.23
6	90.1%	0.31	1.57
7	85.9%	3.40	2.00
8	88.1%	3.86	3.28

## Data Availability

The original contributions presented in this study are included in the article. Further inquiries can be directed to the corresponding author.

## Supplementary Materials

The following supporting information can be downloaded at https://www.mdpi.com/article/10.3390/ijms26157344/s1.
