# Supplementary material for: Informatics-Based Design of Virtual Libraries of Polymer Nano-Composites
Source: Int J Mol Sci. 2025 Jul 30;26(15):7344. doi: 10.3390/ijms26157344 (PMC12347764; doi:10.3390/ijms26157344)
Supplement: Supplementary file 1 [file ijms-26-07344-s001.zip › ijms-3722360-supplementary.pdf]

Supplementary Material

Informatics-Based Design of Virtual Libraries of Polymer Nano-Composites

Qinrui Liu[1] and Scott R. Broderick[1,*]

[1] Department of Materials Design and Innovation, University at Buffalo, Buffalo, NY 14260, USA.

S1. PLS Mathematical Details

PLS finds the maximum variance in the predictor variables (*X*) and finds the correlation factors between *X* and the predicted variables (*Y*) that have maximum variance. In PLS, two linear combinations are generated from the *X* and *Y* respectively and the maximum covariance between *X* and *Y* is calculated. Consider an *X* matrix of size *N*×*K* and an *N*×*M* matrix *Y*. The scores of *X*, t_a (a=1, 2, …, A=the number of PLS components) are calculated as linear combinations of the original variables with the weights w^*_{ka}. The mathematical expression is

$$t_{ia} = \sum_k w^*_{ka} x_{ik} \text{ or } T = XW^* \qquad \text{(S1)}$$

where k=(1, …, K=the number of *X* variables). The predictor variables, *X*, are expressed as:

$$x_{ik} = t_{i1}p^T_{1k} + t_{i2}p^T_{2k} + \cdots + t_{iA}p^T_{Ak} + e_{ik} = \sum_a t_{ia}p_{ak} + e_{ik} \text{ or } X = TP^T + E \qquad \text{(S2)}$$

where e_{ik} is the *X* residuals. Similarly, for predicted variables *Y*, if the scores of *Y* are u_a and the weights c_{am}:

$$y_{im} = \sum_a u_{ia}c_{am} + g_{im} \text{ or } Y = UC^T + G \qquad \text{(S3)}$$

Since scores of *X* are good predictors of *Y* in PLS, then:

$$y_{im} = \sum_a t_{ia} c_{am} + f_{im} \text{ or } Y = TC^T + F \tag{S4}$$

where F represents the error between observed values and the predicted response. Using equation (S1), the equation (S4) is also expressed as

$$y_{im} = \sum_a c_{am} \sum_k w^*_{ka} x_{ik} + f_{im} = \sum_k b_{mk} x_{ik} + f_{im} \text{ or } Y = XW^* C^T + F = XB + F \tag{S5}$$

From equation (S5), the PLS regression coefficients β_{mk} is written as

$$\beta_{mk} = \sum_a c_{am} w^*_{ka} \text{ or } B = W^* C^T \tag{S6}$$

The multidimensional space of *X* is reduced to the A-dimensional hyper plane. Since the scores are good predictors of *Y*, the correlation of *Y* is formed on this hyper plane. The loadings of *X* (*P*) represent the orientation of each of the components of the hyper plane. According to the approach of Phatak and de Jong, after *n* dimensions have been extracted the following equations are available.

$$T_n = XW^*_n, P_n = X^T T_n (T_n^T T_n)^{-1}, W^* = W_n (P_n^T W_n)^{-1} \tag{S7}$$

The prediction of *y* then has a general form:

$$\hat{y}^n_{PLS} = T_n (T_n^T T_n)^{-1} T_n^T y \tag{S8}$$

From the equations (S7), equation (S8) is written as:

$$\hat{y}^n_{PLS} = X\hat{\beta}^n_{PLS} = XW^*_n (W_n^T X^T XW^*_n)^{-1} W_n^T X^T X\hat{\beta}_{OLS} \tag{S9}$$

S.2. Description of Data Organization

The following is a portion of the data used for the analyses in this paper. This table serves to better illustrate the organization of the data and the development and selection of descriptors.

Polymer Matrix	Polyethene	PEEK	Poly(bisphenol A carbonate)	polyether sulfone	Polystrene	PET	poly(hexano-6-lactam)	PEEK
% CNF	10	62	20	20	5	0	0	0
Electrical Conductivity $(ohm*cm)^{-1}$	1.02E-14	1.25E+01	7.00E-07	4.71E-04	2.00E-05	2.93E-16	1.00E-13	2E-18
Strength (GPa)						4.00E-01	6.25E-01	0.3085
Toughness (%)						2.85E+02	2.51E+02	253.05
%C	0.333	0.571	0.485	0.500	0.500	0.455	0.316	0.559
%H	0.667	0.343	0.424	0.407	0.500	0.364	0.579	0.353
%N	0.000	0.000	0.000	0.000	0.000	0.000	0.053	0.000
%O	0.000	0.086	0.091	0.074	0.000	0.182	0.053	0.088
%S	0.000	0.000	0.000	0.019	0.000	0.000	0.000	0.000
BB Amine	0.000	0.000	0.000	0.000	0.000	0.000	0.000	0.000
BB Benzyl	0.000	0.300	0.250	0.286	0.000	0.111	0.000	0.250
BB C	1.000	0.600	0.500	0.643	1.000	0.667	0.857	0.583
BB N	0.000	0.000	0.000	0.000	0.000	0.000	0.143	0.000
BB S	0.000	0.000	0.000	0.071	0.000	0.000	0.000	0.000
BB O	0.000	0.100	0.250	0.000	0.000	0.222	0.000	0.167
NBB Methane	0.000	0.000	0.667	0.500	0.000	0.000	0.000	0.000
NBB O	0.000	1.000	0.333	0.500	0.000	1.000	1.000	1.000
N (C,C,C)	0.000	0.000	0.000	0.000	0.000	0.000	0.000	0.000
N (C,C,H)	0.000	0.000	0.000	0.000	0.000	0.000	0.125	0.000
O (C)	0.000	0.087	0.053	0.063	0.000	0.143	0.125	0.045
O (C,H)	0.000	0.000	0.000	0.000	0.000	0.000	0.000	0.000
O (C,C)	0.000	0.043	0.105	0.063	0.000	0.143	0.000	0.091
C (N,O,C)	0.000	0.000	0.000	0.000	0.000	0.000	0.125	0.000
C (1.5C,1.5C,C)	0.000	0.174	0.105	0.063	0.125	0.143	0.000	0.091
C (1.5C,1.5C,N)	0.000	0.000	0.000	0.000	0.000	0.000	0.000	0.000
C (1.5C,1.5C,O)	0.000	0.087	0.105	0.125	0.000	0.000	0.000	0.182

C (1.5C,1.5C,H)	0.000	0.522	0.421	0.500	0.625	0.286	0.000	0.545
C (C,H,H,H)	0.000	0.000	0.105	0.063	0.000	0.000	0.000	0.000
C (C,C,C,C)	0.000	0.000	0.053	0.031	0.000	0.000	0.000	0.000
C (C,C,O)	0.000	0.087	0.000	0.000	0.000	0.000	0.000	0.045
C (C,O,H,H)	0.000	0.000	0.000	0.000	0.000	0.143	0.000	0.000
C (C,C,O,H)	0.000	0.000	0.000	0.000	0.000	0.000	0.000	0.000
C (C,O,O)	0.000	0.000	0.053	0.000	0.000	0.143	0.000	0.000
C (C,C,H,H)	1.000	0.000	0.000	0.000	2.000	0.000	0.500	0.000
C (C,N,H,H)	0.000	0.000	0.000	0.000	0.000	0.000	0.125	0.000
C (1.5C,1.5C,S)	0.000	0.000	0.000	0.063	0.000	0.000	0.000	0.000
S (C,C)	0.000	0.000	0.000	0.000	0.000	0.000	0.000	0.000
